# Antimicrobial susceptibility of gram-positive and gram-negative bacteria: a 5-year retrospective analysis at a multi-hospital healthcare system in Saudi Arabia

**DOI:** 10.1186/s12941-021-00450-x

**Published:** 2021-06-12

**Authors:** Saad Alhumaid, Abbas Al Mutair, Zainab Al Alawi, Ahmad J. Alzahrani, Mansour Tobaiqy, Ahmed M. Alresasi, Ibrahim Bu-Shehab, Issa Al-Hadary, Naif Alhmeed, Mossa Alismail, Ahmed H. Aldera, Fadhil AlHbabi, Haifa Al-Shammari, Ali A. Rabaan, Awad Al-Omari

**Affiliations:** 1 Administration of Pharmaceutical Care, Alahsa Health Cluster, Ministry of Health, Rashdiah Street, P. O. Box 12944, Alahsa, 31982 Saudi Arabia; 2 Research Center, Almoosa Specialist Hospital, Alahsa, Saudi Arabia; 3 School of Nursing, Wollongong University, Wollongong, Australia; 4 Department of Pediatrics, College of Medicine, King Faisal University, Alahsa, Saudi Arabia; 5 College of Medicine, Al-Imam Mohammed Ibn Saud Islamic University, Riyadh, Saudi Arabia; 6 Department of Pharmacology, College of Medicine, University of Jeddah, Jeddah, Saudi Arabia; 7 Naif Alhmeed, Administration of Supply and Shared Services, Ministry of Health, Riyadh, Saudi Arabia; 8 Pharmacy Department, King Faisal General Hospital, Alahsa, Saudi Arabia; 9 Pharmacy Department, Prince Saud Bin Jalawi Hospital, Alahsa, Saudi Arabia; 10 Virology Department, Regional Laboratory and Blood Bank, Riyadh, Saudi Arabia; 11 Department of Histopathology, King Saud Medical City, Riyadh, Saudi Arabia; 12 Molecular Diagnostics Laboratory, Johns Hopkins Aramco Healthcare, Dhahran, Saudi Arabia; 13 College of Medicine, Alfaisal University, Riyadh, Saudi Arabia; 14 Research Center, Dr. Sulaiman Al Habib Medical Group, Riyadh, Saudi Arabia

**Keywords:** Antibiotics, Antimicrobials, Gram-positive, Gram-negative, Healthcare-associated infections, Rates, Saudi Arabia, Sensitivity, Susceptibility

## Abstract

**Background:**

Studying time-related changes in susceptible pathogens causing healthcare-associated infections (HAIs) is vital in improving local antimicrobial and infection control practices.

**Objectives:**

Describe susceptibility patterns to several antimicrobials in gram-positive and gram-negative pathogens isolated from patients causing HAIs at three private tertiary care hospitals in Saudi Arabia over a 5-year period.

**Methods:**

Data on trends of antimicrobial susceptibility among bacteria causing HAIs events in children and adults at three tertiary private hospitals located in Riyadh and Qassim, Saudi Arabia, were collected retrospectively between 2015 and 2019 using the surveillance data datasets.

**Results:**

Over a 5-year period, 38,624 pathogens caused 17,539 HAI events in 17,566 patients. About 9450 (53.8%) of patients who suffered HAIs were females and the average age was 41.7 ± 14.3 years (78.1% were adults and 21.9% were children). Gram-negative pathogens were 2.3-times more likely to cause HAIs compared to gram-positive bacteria (71.9% vs. 28.1%). The ranking of causative pathogens in decreasing order was: *Escherichia coli* (38%), *Klebsiella* species (15.1%), and *Staphylococcus aureus* (12.6%). Gram-positive isolates were mostly susceptible to linezolid (91.8%) whereas they were resistant to ampicillin (52.6%), cefoxitin (54.2%), and doxycycline (55.9%). Gram-negative isolates were mostly sensitive to tigecycline (95%) whereas they were resistant to cefotaxime (49.5%) and cefixime (59.6%). During the 5 years, there were relatively stable susceptibility patterns to all tested antimicrobials, except for cefotaxime which shown a susceptibility reduction by 41.4%, among *Escherichia coli* and *Klebsiella* species. An increase in the susceptibility of *Acinetobacter* and *Enterobacter* and *Citrobacter* species to all studied antimicrobials was observed except for colistin that had a slight sensitivity reduction in 2019 by 4.3% against *Acinetobacter* species. However, we noted reduced sensitivity of MRSA, CoNS and *Enterococcus* species to gentamicin; and increased resistance of MRSA to linezolid and vancomycin.

**Conclusion:**

The observed increase in susceptibility of gram-positive and gram-negative bacteria to studied antimicrobials is important; however, reduced sensitivity of MRSA, CoNS and *Enterococcus* species to gentamicin; and increased resistance of MRSA to linezolid and vancomycin is a serious threat and calls for effective antimicrobial stewardship programs.

## Background

Antimicrobial resistance (AMR) is a major threat to public health imposing significant health and economic burdens on healthcare system and patients [1, 2]. Unless proactive solutions are found to address AMR, global costs are estimated to reach USD 3 trillion annually by 2050 and an additional 10 million people could die each year; cumulated costs could reach over USD 100 trillion [3]. Decreasing private sector investment in the development of new antimicrobials to treat AMR infections threatens global efforts to fight this danger; and AMR requires international attention and collaboration, because bacteria do not recognize borders. In Saudi Arabia, misuse of antimicrobials is high and complicated primarily because antibiotics are available to buy by anyone over-the-counter via the community pharmacies without a legal prescription [4]. Only two years ago, Saudi Ministry of Health has implemented a nationwide ban on the sale of antibiotics without a legal prescription; however, despite this law, dispensing antibiotics without prescription is still common [4]. Routine clinical microbiology laboratory data provide a profile of the susceptibilities of specific bacteria to antimicrobial agents for monitoring and responding to emerging antimicrobial issues. Data can be utilized to help in the selection of empirical therapy by selecting the most appropriate antibiotics before susceptibility results are available, but remains generally unexploited for purposes of epidemiological surveillance. Although Antimicrobial stewardship programs focus on antibiotic prescribing practice, it is supported by an understanding of local antibiotic susceptibility trends, which in turn depends on the availability of a reliable medical microbiology laboratory resource. The Medical Group has implemented antimicrobial stewardship (AMS) programs since January 2014 and employs various strategies to reduce inappropriate utilization of antimicrobials, minimize the emergence of AMR and lower incidence of health-care-associated infections (HAIs) and reduce cost [1, 5].

Several local studies have estimated the rates of susceptibility among gram-positive and gram-negative bacteria in Saudi Arabia [6–10], but none was comprehensive, and comparisons are complicated by variable methods and study periods that influence the findings explanation and interpretation.

### Aim

This study aimed to examine patterns of antimicrobial susceptibility of gram-positive and gram-negative pathogens isolated from inpatients and outpatients causing HAIs using the surveillance data datasets collected from three HMG hospitals (Altakhassusi, Arryan and Qassim) over a 5-year period, in Saudi Arabia.

### Settings

The private tertiary medical group is considered as one of the largest private healthcare providers in the Middle Eastern region. Currently, the medical group operates 14 medical facilities across Saudi Arabia, UAE and Bahrain, including 7 hospitals and 6 medical centers.

Study was conducted at three tertiary and specialized health facilities with adequate medical professional resources with 237-bed capacity, 365-bed and 150-bed capacity, respectively located in two different cities in Saudi Arabia.

These facilities provide healthcare services to a wide range of patients in various specialties and subspecialties. Yearly, the three healthcare facilities encounter over 127,364 surgical cases, nearly 1,742,144 visits to emergency departments, and over 360,587 admissions.

## Methods

### Study design

Data of trends in antimicrobial susceptibility among of all reports of four types of gram-positive isolates [*Staphylococcus aureus*, Methicillin-resistant *Staphylococcus aureus* (MRSA), Coagulase-negative *staphylococci* (CoNS) and *Enterococcus* species] and six types of gram-negative isolates [*Escherichia coli*, *Klebsiella* species, *Pseudomonas* species, *Acinetobacter* species, *Proteus* species, and *Enterobacter and Citrobacter* species] causing HAIs, collected from the infection control and prevention surveillance data between January 2015 and December 2019 from adult and pediatric patients in three tertiary private hospitals in Saudi Arabia, were extracted using standard customized Excel data collection sheets (Microsoft Corp, Redmond, WA, USA). The antimicrobial susceptibility patterns for selected antimicrobials were analyzed and reported.

We extracted the following patient data from the patient records meeting the inclusion criteria: age, gender, patient location (wards, intensive care units, and outpatient settings), specimen type, HAI type, organism identified, and antimicrobial susceptibility test results.

### Inclusion–exclusion criteria

Data on incidence of targeted bacterial isolates causing HAIs and susceptibility trends of selected pathogens to various antimicrobials collected from medical and surgical wards, intensive care units (ICUs), emergency rooms and hospital-affiliated outpatient clinics from inpatients and outpatients with blood, urinary, rectal, cerebral spinal fluid, respiratory, saliva, nasal, cervical, lavages, wound, tissue, and semen cultures (consecutive, one per patient, per infection site) were included.

Representatives from all clinically important antimicrobial classes have been tested (ampicillin, cloxacillin, amoxicillin/clavulanic acid, piperacillin/tazobactam, cefoxitin, cefazolin, cefuroxime, cefixime, cefotaxime, ceftriaxone, ceftazidime, cefepime, ciprofloxacin, levofloxacin, ofloxacin, nitrofurantoin, erythromycin, clindamycin, trimethoprim-sulfamethoxazole, amikacin, gentamicin, doxycycline, tetracycline, vancomycin, linezolid, imipenem, meropenem, tigecycline and colistin).

Infection events and response of pathogens to antibacterials lacking microorganism and/or culture and sensitivity testing information were excluded.

### Antimicrobial susceptibility testing

Species identification of isolates and their antimicrobial susceptibility profiles were obtained with different automated systems at every single laboratory of the three facilities using (VITEK^®^2 system, BioMariex, France), BD Phoenix system (BD Biosciences, NJ, USA), MicroScan plus (Beckman Coulter, CA, USA), and BD BACTEC system (BD Biosciences) according to manufacturers’ specifications, between 2015 and 2019, with susceptibility interpretations based on the Clinical and Laboratory Standards Institute (CLSI) broth microdilution and breakpoint criteria [11]. To ensure data compatibility, quality control was performed using control strains from the following American Type Culture Collection (ATCCs): *Staphylococcus aureus* ATCC 29213, *Pseudomonas aeruginosa* ATCC 2853, *Escherichia coli* ATCC 25922, *Escherichia coli* ATCC 35218, *Klebsiella pneumoniae* ATCC 27736 and *Enterococcus faecalis* ATCC 29212. Data are only included when the quality control test results were in acceptable ranges.

### Statistical analysis

Variables that were continuous were presented as means and categorical variables were presented as frequencies and percentages. Susceptibility patterns of pathogens were presented over time. The difference in sensitivity trends between 2015 and 2019 was examined using the multivariate analysis of variance (MANOVA) and a two-sided P-values < 0.05 were considered to be statistically significant. The proportion of susceptible isolates was calculated as the sum of susceptible organisms (neither intermediately susceptible nor resistant) relative to the total number of organisms tested. SPSS (Version 25.0. Armonk, NY: IBM Corp) and Microsoft Excel Professional Plus 2019 (Microsoft Corp., Redmond, WA, USA) were used for all statistical analyses.

Our study was performed in accordance with the ethical standards of the Declaration of Helsinki and its later amendments or comparable ethical standards. Ethics approval (RC20.10.95-2) was obtained by the Ethics Committee of the coordinator center (IRB Committee of Dr. Sulaiman Al Habib Medical Group, Riyadh, Saudi Arabia).

## Results

### Incidence of pathogens causing HAIs and patient characteristics

**Fig. 1:**
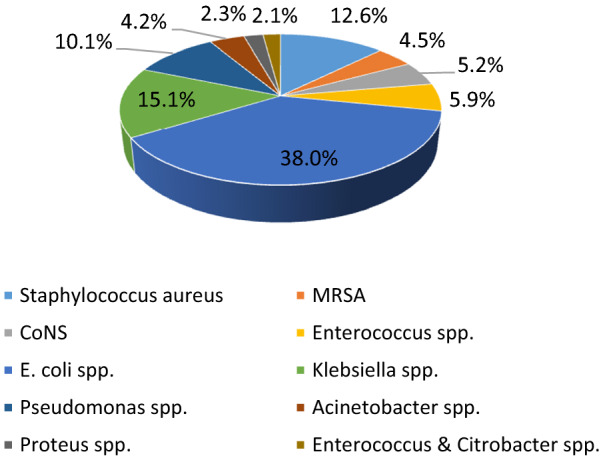
Total frequency of isolated gram-positive and gram-negative bacteria causing healthcare-associated infections in the three facilities in Saudi Arabia (2015–2019)

A total of 41,813 pathogens were isolated over 5 years in the three of our medical group’s facilities of which 38,624 pathogens caused 17,539 HAI events in 17,566 patients. These HAIs events were contracted in HMG Hospital in Altakhassusi (6016 HAI events = 34.3%), HMG Hospital in Arryan (5893 HAI events = 33.6%) and HMG Hospital in Qassim (5630 HAI events = 32.1%). Reported HAIs varied in type: catheter-associated urinary tract infection (CAUTI) (29.4%), central line-associated bloodstream infection (CLABSI) (27.3%), surgical site infection (SSI) (26.1%) and ventilator-associated events (VAE) (17.2%). Processed samples were blood (24.7%), urinary (19.1%), respiratory (13.4%), cerebral spinal fluid (8.5%), cervical (8.2%), saliva (5.2%), nasal (5.1), rectal (4.9%), lavages (4.7%), wound (3.9%), tissue (1.4%), and semen (0.9%). These HAI events were isolated in the intensive care units (37.2%), wards (32.9%), and outpatients (29.9%). In our study, we excluded 6232 (16.1%) HAI events due to the lack of data on the antimicrobial, pathogen, and/or culture response and sensitivity testing. About 9450 (53.8%) of patients who suffered HAIs were identified as females and had a mean age of 41.7 ± 14.3 years (78.1% were adults and 21.9% were children). Of 38,624 isolates taken from clinical specimens between 2015 and 2019, 27,754 (71.9%) were gram-negative organisms and 10,870 (28.1%) were gram-positive organisms. The ranking of causative pathogens in decreasing order was: *Escherichia coli* (38%), *Klebsiella* species (15.1%), *Staphylococcus aureus* (12.6%), *Pseudomonas* species (10.1%), and *Enterococcus* species (5.9%) (Fig. 1).

### Trends of susceptibility among gram-positive bacteria

A total of 79,280 gram-positive pathogen sensitivity events against 14 clinically important antimicrobials occurred at HMG Hospital in Altakhassusi (38.2%), HMG Hospital in Arryan (36.6%), and HMG Hospital in Qassim (25.2%). Gram-positive bacteria showed an overall susceptibility of ≥ 52.6%. Antimicrobial susceptibility patterns in gram-positive pathogens over time are presented in Table 1.

**Table 1:** Antimicrobial susceptibility rates found in gram-positive bacteria causing healthcare-associated infections in three HMG facilities in Saudi Arabia (2015–2019)

*Staphylococcus aureus*	2015			2016			2017			2018			2019			Total			P-value*
	(*N* = 903 isolates)			(*N* = 666 isolates)			(*N* = 940 isolates)			(*N* = 1,122 isolates)			(*N* = 1,222 isolates)			(*N* = 4,853 isolates)			
	T	S	S%	T	S	S%	T	S	S%	T	S	S%	T	S	S%	T	S	S%	
AMP	191	64	33.5	131	52	39.7	169	93	55	157	87	55.4	222	63	28.4	870	359	41.3	0.828
CLX	677	544	80.4	444	331	74.5	797	656	82.3	747	679	90.9	887	735	82.9	3552	2945	82.9	0.649
CTN	230	109	47.4	209	89	42.6	266	134	50.4	266	154	57.9	252	161	63.9	1223	647	52.9	0.937
CIP	664	457	68.8	446	321	72	671	498	74.2	511	453	88.6	548	487	88.9	2840	2216	78	0.993
LVX	389	288	74	228	169	74.1	552	426	77.2	588	532	90.5	700	627	89.6	2457	2042	83.1	0.864
DCN	322	153	47.5	155	87	56.1	181	93	51.4	258	124	48.1	192	139	72.4	1108	596	53.8	0.983
TCN	514	305	59.3	489	423	86.5	891	838	94.1	1001	989	98.8	1201	1079	89.8	4096	3634	88.7	0.335
GMN	888	819	92.2	641	582	90.8	889	871	98	1022	955	93.4	1193	1098	92	4633	4325	93.4	0.775
EMN	722	696	96.4	558	473	84.8	791	740	93.6	971	813	83.7	991	929	93.7	4033	3651	90.5	0.659
CMN	804	780	97	576	513	89.1	896	871	97.2	1103	967	87.7	1187	1071	90.2	4566	4202	92	0.697
NFT	441	317	71.9	334	207	62	454	371	81.7	454	321	70.7	345	266	77.1	2028	1482	73.1	0.712
TMP-SMZ	595	463	77.8	591	524	88.7	865	819	94.7	1022	947	92.7	1201	996	82.9	4274	3749	87.7	0.542
LZD	903	903	100	641	625	97.5	790	773	97.8	1110	1003	90.4	1100	1076	97.8	4544	4380	96.4	0.931
VMN	901	895	99.3	651	624	95.9	938	931	99.3	1113	1109	99.6	1200	1181	98.4	4803	4740	98.7	0.721

MRSA	2015			2016			2017			2018			2019			Total			P-value*
	(*N* = 359 isolates)			(*N* = 335 isolates)			(*N* = 284 isolates)			(*N* = 443 isolates)			(*N* = 310 isolates)			(*N* = 1,731 isolates)			
	T	S	S%	T	S	S%	T	S	S%	T	S	S%	T	S	S%	T	S	S%	
AMP	90	13	14.4	44	8	18.2	51	7	13.7	74	15	20.3	69	21	30.4	328	64	19.5	0.814
CLX	116	72	62.1	147	86	58.5	147	81	55.1	311	138	44.4	290	141	48.6	1011	518	51.2	0.963
CTN	11	7	63.6	33	9	27.3	64	13	20.3	67	11	16.4	50	8	16	225	48	21.3	0.956
CIP	190	141	74.2	187	112	59.9	229	108	47.2	401	198	49.4	301	245	81.4	1308	804	61.5	0.924
LVX	55	31	56.4	99	46	46.5	187	75	40.1	366	131	35.8	260	115	44.2	967	398	41.2	0.693
DCN	222	110	49.5	189	122	64.6	280	141	50.4	410	191	46.6	307	154	50.2	1408	718	51	0.964
TCN	141	101	71.6	211	118	55.9	179	108	60.3	440	251	57	303	271	89.4	1274	849	66.6	0.860
GMN	291	121	41.6	277	129	46.6	151	116	76.8	432	216	50	300	284	94.7	1451	866	59.7	0.888
EMN	144	91	63.2	177	102	57.6	111	97	87.4	331	176	53.2	271	232	85.6	1034	698	67.5	0.878
CMN	220	93	42.3	254	113	44.5	161	108	67.1	411	203	49.4	300	264	88	1346	781	58	0.838
NFT	181	117	64.6	311	213	68.5	281	241	85.8	441	399	90.5	308	287	93.2	1522	1257	82.6	0.346
TMP-SMZ	233	88	37.8	268	114	42.5	229	108	47.2	409	201	49.1	290	227	78.3	1429	738	51.6	0.892
LZD	241	118	49	264	138	52.3	223	123	55.2	439	251	57.2	299	175	58.5	1466	805	54.9	0.974
VMN	299	121	40.5	285	138	48.4	258	123	47.7	440	251	57	305	175	57.4	1587	808	50.9	0.975

CoNS	2015			2016			2017			2018			2019			Total			P-value*
	(*N* = 543 isolates)			(*N* = 276 isolates)			(*N* = 351 isolates)			(*N* = 426 isolates)			(*N* = 424 isolates)			(*N* = 2,020 isolates)			
	T	S	S%	T	S	S%	T	S	S%	T	S	S%	T	S	S%	T	S	S%	
AMP	190	31	16.3	177	21	11.9	166	33	19.9	291	42	14.4	348	51	14.7	1172	178	15.2	0.948
CLX	164	88	53.7	190	64	33.7	289	96	33.2	408	146	35.8	333	114	34.2	1384	508	36.7	0.936
CTN	491	263	53.6	270	239	99.6	340	301	88.5	420	364	86.7	409	373	91.2	1930	1570	81.3	0.962
CIP	277	122	44	257	124	48.2	300	94	31.3	360	129	35.8	340	170	50	1534	639	41.7	0.991
LVX	130	46	35.4	211	56	26.5	288	142	49.3	420	173	41.2	211	85	40.3	1260	502	39.8	0.903
DCN	511	336	65.8	271	261	96.3	349	313	89.7	410	378	92.2	420	381	90.7	1961	1669	85.1	0.951
TCN	499	169	33.9	270	211	78.1	333	283	85	420	316	75.2	424	378	89.2	1946	1357	69.7	0.930
GMN	538	292	54.3	255	176	69	348	269	77.3	418	320	76.6	420	327	77.9	1979	1384	69.9	0.982
EMN	333	95	28.5	116	51	44	270	83	30.7	339	106	31.3	198	98	49.5	1256	433	34.5	0.966
CMN	531	255	48	266	156	58.6	269	235	87.4	399	282	70.7	339	293	86.4	1804	1221	67.7	0.981
NFT	170	77	45.3	233	99	42.5	331	225	68	411	250	60.8	400	344	86	1545	995	64.4	0.819
TMP-SMZ	369	153	41.5	276	199	72.1	350	298	85.1	400	349	87.3	407	327	80.3	1802	1326	73.6	0.831
LZD	541	535	98.9	276	273	98.9	351	351	100	426	426	100	424	424	100	2018	2009	99.6	0.967
VMN	543	537	98.9	276	276	100	351	351	100	426	426	100	424	424	100	2020	2014	99.7	0.968

*Enterococcus* species	2015			2016			2017			2018			2019			Total			P-value*
	(*N* = 481 isolates)			(*N* = 370 isolates)			(*N* = 447 isolates)			(*N* = 411 isolates)			(*N* = 557 isolates)			(*N* = 2266 isolates)			
	T	S	S%	T	S	S%	T	S	S%	T	S	S%	T	S	S%	T	S	S%	
AMP	460	305	66.3	330	252	76.4	422	378	89.6	377	318	84.4	533	508	95.3	2122	1761	83	0.539
CLX	469	280	59.7	340	299	87.9	430	364	84.7	388	381	98.2	499	433	86.8	2126	1757	82.6	0.618
CTN	281	61	21.7	277	79	28.5	414	110	26.6	350	124	35.4	444	151	34	1766	525	29.7	0.850
CIP	369	164	44.4	299	127	42.5	398	169	42.5	322	111	34.5	471	97	20.6	1859	668	35.9	0.965
LVX	379	243	64.1	310	182	58.7	441	305	69.2	401	275	68.6	333	188	56.5	1864	1193	64	0.928
DCN	244	32	13.1	177	20	11.3	288	44	15.3	200	59	29.5	347	67	19.3	1256	222	17.7	0.812
TCN	201	32	15.9	222	40	18	333	203	61	190	92	48.4	500	143	28.6	1446	510	35.3	0.213
GMN	298	113	37.9	191	76	39.8	430	123	28.6	310	152	49	499	183	36.7	1728	647	37.4	0.972
EMN	411	145	35.3	301	42	14	357	153	42.9	188	53	28.2	331	65	19.6	1588	458	28.8	0.824
CMN	177	28	15.8	191	45	23.6	299	61	20.4	191	77	40.3	189	81	42.9	1047	292	27.9	0.506
NFT	322	116	36	355	300	84.5	438	393	89.7	391	364	93.1	541	482	89.1	2047	1655	80.9	0.306
TMP-SMZ	280	63	22.5	339	276	81.4	409	291	71.1	336	273	81.3	534	376	70.4	1898	1279	67.4	0.646
LZD	481	470	97.7	370	370	100	447	447	100	411	411	100	557	556	99.8	2266	2254	99.5	0.874
VMN	481	237	49.3	370	361	97.6	440	435	98.9	410	364	88.8	550	537	97.6	2251	1934	85.9	0.517

Overall	2015			2016			2017			2018			2019			Total			P-value*
	(*N* = 2286 isolates)			(*N* = 1647 isolates)			(*N* = 2022 isolates)			(*N* = 2402 isolates)			(*N* = 2513 isolates)			(*N* = 10,870 isolates)			
	T	S	S%	T	S	S%	T	S	S%	T	S	S%	T	S	S%	T	S	S%	
AMP	931	413	44.4	682	333	48.8	808	511	63.2	899	462	51.4	1172	643	54.9	4492	2362	52.6	0.971
CLX	1426	984	69	1121	780	69.6	1663	1197	72	1854	1344	72.5	2009	1423	70.8	8073	5728	71	0.874
CTN	1013	440	43.4	789	446	56.5	1084	558	51.5	1103	653	59.2	1155	693	60	5144	2790	54.2	0.933
CIP	1500	884	58.9	1189	684	57.5	1598	869	54.4	1594	891	55.9	1660	999	60.2	7541	4327	57.4	0.969
LVX	953	608	63.8	848	453	53.4	1468	948	64.6	1775	1111	62.6	1504	1015	67.5	6548	4135	63.1	0.610
DCN	1299	631	48.6	792	490	61.9	1098	591	53.8	1278	752	58.8	1266	741	58.5	5733	3205	55.9	0.939
TCN	1355	607	44.8	1192	792	66.4	1736	1432	82.5	2051	1648	80.4	2428	1871	77.1	8762	6350	72.5	0.556
GMN	2015	1345	66.7	1364	963	70.6	1818	1379	75.9	2182	1643	75.3	2412	1892	78.4	9791	7222	73.8	0.905
EMN	1610	1027	63.8	1152	668	58	1529	1073	70.2	1829	1148	62.8	1791	1324	73.9	7911	5240	66.2	0.965
CMN	1732	1156	66.7	1287	827	64.3	1625	1275	78.5	2104	1529	72.7	2015	1709	84.8	8763	6496	74.1	0.922
NFT	1114	627	56.3	1233	819	66.4	1504	1230	81.8	1697	1334	78.6	1594	1379	86.5	7142	5389	75.5	0.032
TMP-SMZ	1477	767	51.9	1474	1113	75.5	1853	1516	81.8	2167	1770	81.7	2432	1926	79.2	9403	7092	75.4	0.595
LZD	2166	2026	93.5	1551	1406	90.7	1811	1694	93.5	2386	2091	87.6	2380	2231	93.7	10,294	9448	91.8	0.875
VMN	2224	1790	80.5	1582	1399	88.4	1987	1849	92.6	2389	2163	90	2479	2357	93.5	10,661	9558	89.1	0.901

*N* Number of pathogens causing healthcare-associated infections, *T* number of tested isolates, *S* number of susceptible pathogens, *S* (%) percentage of susceptible pathogens, *MRSA* methicillin-resistant *Staphylococcus aureus*, *CoNS* coagulase-negative *staphylococci*, *AMP* ampicillin, *CLX* cloxacillin, *CTN* cefoxitin, *CIP* ciprofloxacin, *LVX* levofloxacin, *NFT* nitrofurantoin, *EMN* erythromycin, *CMN* clindamycin, *TMP-SMZ* trimethoprim-sulfamethoxazole, *GMN* gentamicin, *DCN* doxycycline, *TCN* tetracycline, *VMN* vancomycin, *LZD* linezolid

*Multivariate analysis of variance (MANOVA) for resistance trend

Generally, the highest susceptibilities of gram-positive pathogens to antimicrobials were seen towards vancomycin and linezolid by *Staphylococcus aureus*, 98.7% and 96.4%; CoNS, 99.7% and 99.6%; and *Enterococcus* species, 99.5% and 85.9%; respectively. Moreover, *Staphylococcus aureus* was found to be highly sensitive to gentamicin (93.4%), clindamycin (92%), and erythromycin (90.5%); MRSA was most sensitive to nitrofurantoin (82.6%); CoNS was sensitive to doxycycline (85.1%) and cefoxitin (81.3%); and *Enterococcus* species was sensitive to ampicillin (83%), cloxacillin (82.6%) and nitrofurantoin (80.9%) over the 5-year period.

In opposite, lowest susceptibilities of gram-positive pathogens to antimicrobials were seen to ampicillin by CoNS, 15.2%; MRSA, 19.5%; and *Staphylococcus aureus*, 41.3%; respectively. Also, *Enterococcus* species was least susceptible to doxycycline (17.7%); and MRSA was slightly sensitive to cefoxitin (21.3%).

Tetracycline, trimethoprim-sulfamethoxazole, levofloxacin and cloxacillin retained activity against 88.7%, 87.7%, 83.1%, and 82.9% of *Staphylococcus aureus* isolates, respectively, whereas trimethoprim-sulfamethoxazole was active against 73.6% of the CoNS isolates.

Over the 5 years, sensitivity of nitrofurantoin to overall gram-positive bacteria was the only antimicrobial to increase significantly (30.2% increase, p-value = 0.032). Prominent insignificant increase in the susceptibility of specific gram-positive bacteria to some antimicrobials occurred in 2019 compared to 2015 by: 30.5% for *Staphylococcus aureus* to tetracycline; 53.1% and 45.7% for MRSA to gentamicin and clindamycin, respectively; 37.6%, 55.3%,38.4%, 38.5% and 40.7% for CoNS to cefoxitin, tetracycline, clindamycin, trimethoprim-sulfamethoxazole and nitrofurantoin, respectively; 53.1%, 47.9% and 48.3% for *Enterococcus* species to trimethoprim-sulfamethoxazole, nitrofurantoin and vancomycin, respectively. However, noticeable insignificant decrease in susceptibility were seen in 2019 compared to 2015 by: 47.6% for MRSA to cefoxitin; and 23.8% for *Enterococcus* species to ciprofloxacin.

Overall, among the studied antibiotics the gram-positive isolates were mostly sensitive to linezolid (91.8%) whereas they were resistant to ampicillin (52.6%), cefoxitin (54.2%), and doxycycline (55.9%) (Table 1).

### Trends of susceptibility among gram-negative bacteria

A total of 314,624 gram-negative pathogen sensitivity events against 21 clinically important antimicrobials occurred at HMG Hospital in Altakhassusi (35.9%), HMG Hospital in Arryan (39.3%), and HMG Hospital in Qassim (24.8%). Gram-negative bacteria showed an overall susceptibility of ≥ 49.5%. Antimicrobial susceptibility patterns in gram-negative pathogens over time are presented in Table 2.

**Table 2:** Antimicrobial susceptibility rates found in gram-negative bacteria causing healthcare-associated infections in three HMG facilities in Saudi Arabia (2015–2019)

*Escherichia coli*	2015			2016			2017			2018			2019			Total			P-value*
	(*N* = 2,481 isolates)			(*N* = 2,458 isolates)			(*N* = 2,509 isolates)			(*N* = 3,085 isolates)			(*N* = 4,149 isolates)			(*N* = 14,682 isolates)			
	T	S	S%	T	S	S%	T	S	S%	T	S	S%	T	S	S%	T	S	S%	
AMP	1100	761	69.2	1130	793	70.2	1158	803	69.3	1100	987	89.7	1800	1654	91.9	6288	4998	79.5	0.460
AMX/CLA	1920	1679	87.4	2100	1805	86	2103	1874	89.1	2700	2326	86.1	3258	2825	86.7	12,081	10,509	87	0.835
CZN	333	126	37.8	999	841	84.2	666	470	70.6	987	631	63.9	1852	1412	76.2	4837	3480	71.9	0.683
CRX	788	660	83.8	1125	828	73.6	547	378	69.1	981	732	74.6	1987	1505	75.7	5428	4103	75.6	0.911
CFX	1968	819	41.6	645	496	76.9	752	432	57.4	896	772	86.2	879	534	60.8	5140	3053	59.4	0.981
CTM	456	253	55.5	488	282	57.8	456	238	52.2	541	290	53.6	654	274	41.9	2595	1337	51.5	0.920
CTX	2388	1764	73.9	1933	1757	90.9	1901	1610	84.7	2456	2134	86.9	3896	3007	77.2	12,574	10,272	81.7	0.682
CZM	1963	1059	53.9	1025	896	87.4	2005	1113	55.5	1754	1275	72.7	1785	1384	77.5	8532	5727	67.1	0.998
CPM	1347	1007	74.8	1987	1775	89.3	2111	1764	83.6	2666	2158	80.9	3101	2849	91.9	11,212	9553	85.2	0.846
IPM	2477	2451	99	2358	2158	91.5	2430	2395	98.6	3081	3069	99.6	4130	4022	97.4	14,476	14,095	97.4	0.676
MPM	2480	2412	97.3	2400	2238	93.3	2414	2392	99.1	3074	3048	99.2	4099	4022	98.1	14,467	14,112	97.5	0.654
PIP-TZP	2360	2270	96.2	2347	2146	91.4	2456	2322	94.5	2996	2882	96.2	3896	3533	90.7	14,055	13,153	93.6	0.805
TMP-SMZ	1456	1293	88.8	1745	1463	83.8	1736	1249	71.9	1987	1775	89.3	2898	2589	89.3	9822	8369	85.2	0.529
GMN	2223	2128	95.7	2314	2178	94.1	2300	2124	92.3	2965	2742	92.5	3991	3589	89.9	13,793	12,761	92.5	0.726
ACN	2470	2135	86.4	2389	2400	100.5	2490	2414	96.9	3030	3009	99.3	4110	4030	98.1	14,489	13,988	96.5	0.697
CIP	2001	1726	86.3	1999	1804	90.2	1800	1737	96.5	2789	2240	80.3	3008	2951	98.1	11,597	10,458	90.2	0.739
OXN	1136	785	69.1	1107	909	82.1	700	612	87.4	698	548	78.5	687	442	64.3	4328	3296	76.2	0.967
LVX	1147	785	68.4	1165	918	78.8	565	438	77.5	1365	1016	74.4	1777	1393	78.4	6019	4550	75.6	0.923
NFT	1455	1241	85.3	2411	2317	96.1	2425	2314	95.4	2905	2704	93.1	3896	3790	97.3	13,092	12,366	94.5	0.489
TGN	2377	2269	95.5	2450	2420	98.8	2500	2499	100	3083	3069	99.5	4144	4131	99.7	14,554	14,388	98.9	0.420
CLN	1365	1106	81	1130	722	63.9	789	570	72.2	2029	1016	50.1	4011	3974	99.1	9324	7388	79.2	0.120

*Klebsiella* species	2015			2016			2017			2018			2019			Total			P-value*
	(*N* = 1,000 isolates)			(*N* = 839 isolates)			(*N* = 1,299 isolates)			(*N* = 1,357 isolates)			(*N* = 1,356 isolates)			(*N* = 5,851 isolates)			
	T	S	S%	T	S	S%	T	S	S%	T	S	S%	T	S	S%	T	S	S%	
AMP	300	206	68.7	222	172	77.5	221	94	42.5	801	611	76.3	512	333	65	2056	1416	68.9	0.258
AMX/CLA	654	534	81.7	654	565	86.4	906	841	92.8	1001	938	93.7	896	739	82.5	4111	3617	88	0.772
CZN	159	75	47.2	400	312	78	456	356	78.1	562	467	83.1	753	462	61.4	2330	1672	71.8	0.786
CRX	654	223	34.1	258	137	53.1	358	278	77.7	452	354	78.3	963	369	38.3	2685	1361	50.7	0.975
CFX	700	444	63.4	402	321	79.9	456	295	64.7	458	370	80.8	456	269	59	2472	1699	68.7	0.989
CTM	112	82	73.2	111	86	77.5	255	104	40.8	320	134	41.9	358	114	31.8	1156	520	45	0.997
CTX	753	543	72.1	654	507	77.5	801	770	96.1	987	887	89.9	1,196	851	71.2	4391	3558	81	0.607
CZM	656	367	55.9	456	326	71.5	687	413	60.1	454	394	86.8	756	396	52.4	3009	1896	63	0.269
CPM	550	310	56.4	654	589	90.1	1010	845	83.7	900	829	92.1	1120	814	72.7	4234	3387	80	0.821
IPM	961	778	81	832	781	93.9	1190	1113	93.5	1290	1208	93.6	1310	1116	85.2	5583	4996	89.5	0.626
MPM	800	742	92.8	765	731	95.6	1120	1104	98.6	1291	1207	93.5	1312	1104	84.1	5288	4888	92.4	0.544
PIP-TZP	751	631	84	741	664	89.6	1130	933	82.6	1140	1021	89.6	1100	906	82.4	4862	4155	85.5	0.765
TMP-SMZ	654	535	81.8	659	596	90.4	874	788	90.2	1122	903	80.5	1145	905	79	4454	3727	83.7	0.561
GMN	963	696	72.3	801	700	87.4	1122	1019	90.8	1260	1167	92.6	1258	1152	91.6	5404	4734	87.6	0.462
ACN	852	774	90.8	811	782	96.4	1125	1087	96.6	1290	1150	89.1	1322	1272	96.2	5400	5065	93.8	0.604
CIP	789	652	82.6	753	686	91.1	1100	954	86.7	1299	1068	82.2	1300	916	70.5	5241	4276	81.6	0.641
OXN	350	265	75.7	402	273	67.9	582	404	69.4	654	589	90.1	1101	789	71.7	3089	2320	75.1	0.624
LVX	300	265	88.3	582	321	55.2	452	360	79.6	542	441	81.4	900	745	82.8	2776	2132	76.8	0.870
NFT	258	137	53.1	456	322	70.6	652	505	77.5	521	478	91.7	456	379	83.1	2343	1821	77.7	0.106
TGN	753	512	68	500	356	71.2	587	417	71	530	517	97.5	874	639	73.1	3244	2441	75.2	0.984
CLN	466	378	81.1	665	654	98.3	1200	1179	98.3	529	517	97.7	1199	1023	85.3	4059	3751	92.4	0.736

*Pseudomonas* species	2015			2016			2017			2018			2019			Total			P-value*
	(*N* = 850 isolates)			(*N* = 806 isolates)			(*N* = 696 isolates)			(*N* = 733 isolates)			(*N* = 837 isolates)			(*N* = 3,922 isolates)			
	T	S	S%	T	S	S%	T	S	S%	T	S	S%	T	S	S%	T	S	S%	
AMP	221	46	20.8	400	193	48.3	333	269	80.8	401	377	94	514	441	85.8	1869	1326	70.9	0.636
AMX/CLA	410	296	72.2	147	78	53.1	415	300	72.3	159	94	59.1	652	416	63.8	1783	1184	66.4	0.519
CZN	119	61	51.3	302	111	36.8	512	264	51.6	412	303	73.5	452	329	72.8	1797	1068	59.4	0.046
CRX	300	109	36.3	411	178	43.3	466	222	47.6	454	301	66.3	552	489	88.6	2183	1299	59.5	0.188
CFX	147	76	51.7	430	188	43.7	501	249	49.7	541	334	61.7	611	401	65.6	2230	1248	56	0.186
CTM	109	29	26.6	156	67	42.9	247	110	44.5	268	137	51.1	254	133	52.4	1034	476	46	0.923
CTX	358	276	77.1	547	369	67.5	240	110	45.8	260	137	52.7	219	133	60.7	1624	1025	63.1	0.746
CZM	800	676	84.5	789	686	86.9	614	495	80.6	660	480	72.7	653	558	85.5	3516	2895	82.3	0.952
CPM	598	300	50.2	658	567	86.2	611	512	83.8	614	500	81.4	678	568	83.8	3159	2447	77.5	0.961
IPM	753	541	71.8	782	614	78.5	660	574	87	603	576	95.5	801	710	88.6	3599	3015	83.8	0.925
MPM	741	564	76.1	788	617	78.3	671	570	84.9	654	582	89	822	713	86.7	3676	3046	82.9	0.944
PIP-TZP	820	631	77	799	692	86.6	680	590	86.8	721	608	84.3	810	742	91.6	3830	3263	85.2	0.956
TMP-SMZ	658	321	48.8	654	456	69.7	514	477	92.8	654	522	79.8	769	687	89.3	3249	2463	75.8	0.535
GMN	830	744	89.6	800	730	91.3	688	642	93.3	701	672	95.9	780	759	97.3	3799	3547	93.4	0.989
ACN	840	755	89.9	801	756	94.4	680	665	97.8	699	686	98.1	820	784	95.6	3840	3646	94.9	0.990
CIP	801	631	78.8	771	646	83.8	670	604	90.1	652	597	91.6	700	667	95.3	3594	145	87.5	0.997
OXN	80	13	16.3	154	71	46.1	230	110	47.8	325	187	57.5	400	191	47.8	1189	572	48.1	0.030
LVX	775	608	78.5	658	492	74.8	677	369	54.5	541	359	66.4	521	381	73.1	3172	2209	69.6	0.921
NFT	302	111	36.8	321	128	39.9	321	177	55.1	428	201	47	451	290	64.3	1823	907	49.8	0.491
TGN	346	339	98	420	409	97.4	511	498	97.5	541	533	98.5	681	678	99.6	2499	2457	98.3	0.142
CLN	711	706	99.3	670	661	98.7	587	571	97.3	301	291	96.7	328	321	97.9	2597	2550	98.2	0.853

*Acinetobacter* species	2015			2016			2017			2018			2019			Total			P-value*
	(*N* = 611 isolates)			(*N* = 275 isolates)			(*N* = 280 isolates)			(*N* = 262 isolates)			(*N* = 182 isolates)			(*N* = 1,610 isolates)			
	T	S	S%	T	S	S%	T	S	S%	T	S	S%	T	S	S%	T	S	S%	
AMP	90	33	36.7	150	74	49.3	320	110	34.4	264	130	49.2	275	142	51.6	1099	489	44.5	0.262
AMX/CLA	110	29	26.4	120	53	44.2	168	76	45.2	191	91	47.6	201	103	51.2	790	352	44.6	0.291
CZN	60	19	31.7	60	21	35	96	29	30.2	67	34	50.7	79	44	55.7	362	147	40.6	0.538
CRX	75	24	32	82	36	43.9	78	33	42.3	86	41	47.7	98	53	54.1	419	187	44.6	0.480
CFX	64	11	17.2	70	24	34.3	77	23	29.9	76	36	47.4	88	47	53.4	375	141	37.6	0.101
CTM	58	10	17.2	35	10	28.6	34	12	35.3	39	17	43.6	47	26	55.3	213	75	35.2	0.551
CTX	86	18	20.9	31	14	45.2	33	22	66.7	71	29	40.8	76	38	50	297	121	40.7	0.478
CZM	64	14	21.9	43	21	48.8	79	33	41.8	98	46	46.9	98	51	52	382	165	43.2	0.523
CPM	23	3	13	29	11	37.9	39	17	43.6	70	36	51.4	81	43	53.1	242	110	45.5	0.573
IPM	240	62	25.8	78	35	44.9	108	50	46.3	130	70	53.8	157	79	50.3	713	296	41.5	0.450
MPM	149	35	23.5	127	62	48.8	100	50	50	129	70	54.3	171	91	53.2	676	308	45.6	0.112
PIP-TZP	97	17	17.5	89	44	49.4	70	38	54.3	90	47	52.2	110	59	53.6	456	205	45	0.550
TMP-SMZ	310	75	24.2	348	172	49.4	287	141	49.1	220	115	52.3	300	177	59	1465	680	46.4	0.420
GMN	378	93	24.6	186	144	77.4	190	88	46.3	171	91	53.2	240	127	52.9	1165	543	46.6	0.660
ACN	220	49	22.3	189	84	44.4	121	56	46.3	155	80	51.6	168	94	56	853	363	42.6	0.413
CIP	127	29	22.8	114	49	43	130	59	45.4	125	67	53.6	148	78	52.7	644	282	43.8	0.415
OXN	89	21	23.6	94	36	38.3	111	48	43.2	122	67	54.9	139	73	52.5	555	245	44.1	0.322
LVX	40	8	20	17	8	47.1	36	17	47.2	40	24	60	58	33	56.9	191	90	47.1	0.697
NFT	128	41	32	321	77	24	310	140	45.2	290	151	52.1	287	151	52.6	1336	560	41.9	0.102
TGN	369	350	94.9	197	188	95.4	142	139	97.9	148	138	93.2	113	108	95.6	969	923	95.3	0.022
CLN	401	381	95	134	124	92.5	169	164	97	120	116	96.7	129	117	90.7	953	902	94.6	0.089

*Proteus* species	2015			2016			2017			2018			2019			Total			P-value*
	(*N* = 212 isolates)			(*N* = 192 isolates)			(*N* = 150 isolates)			(*N* = 135 isolates)			(*N* = 197 isolates)			(*N* = 886 isolates)			
	T	S	S%	T	S	S%	T	S	S%	T	S	S%	T	S	S%	T	S	S%	
AMP	200	92	46	186	89	47.8	74	40	54.1	99	59	59.6	181	100	55.2	740	380	51.4	0.527
AMX/CLA	201	143	71.1	187	146	78.1	147	100	68	129	114	88.4	170	155	91.2	834	658	78.9	0.797
CZN	107	48	44.9	180	99	55	140	126	90	125	101	80.8	190	132	69.5	742	506	68.2	0.828
CRX	83	35	42.2	178	44	24.7	91	51	56	113	74	65.5	130	81	62.3	595	285	47.9	0.863
CFX	60	19	31.7	62	27	43.5	89	49	55.1	128	77	60.2	191	110	57.6	530	282	53.2	0.266
CTM	112	36	32.1	81	40	49.4	78	52	66.7	97	64	66	141	76	53.9	509	268	52.7	0.760
CTX	203	112	55.2	179	133	74.3	130	121	93.1	127	100	78.7	180	161	89.4	819	627	76.6	0.878
CZM	150	77	51.3	150	70	46.7	136	115	84.6	131	79	60.3	174	116	66.7	741	457	61.7	0.913
CPM	90	41	45.6	128	63	49.2	137	71	51.8	131	118	90.1	179	127	70.9	665	420	63.2	0.692
IPM	130	61	46.9	167	78	46.7	141	102	72.3	133	127	95.5	160	140	87.5	731	508	69.5	0.619
MPM	200	121	60.5	189	150	79.4	149	130	87.2	121	101	83.5	189	179	94.7	848	681	80.3	0.821
PIP-TZP	199	113	56.8	180	129	71.7	147	122	83	129	119	92.2	179	161	89.9	834	644	77.2	0.935
TMP-SMZ	131	64	48.9	179	78	43.6	140	98	70	118	100	84.7	189	109	57.7	757	449	59.3	0.598
GMN	141	88	62.4	167	109	65.3	145	140	96.6	120	102	85	170	148	87.1	743	587	79	0.417
ACN	198	115	58.1	169	138	81.7	140	131	93.6	129	117	90.7	181	173	95.6	817	674	82.5	0.871
CIP	191	93	48.7	187	108	57.8	135	133	98.5	131	122	93.1	179	147	82.1	823	603	73.3	0.775
OXN	76	30	39.5	71	39	54.9	70	40	57.1	109	61	56	131	77	58.8	457	247	54	0.588
LVX	40	17	42.5	40	22	55	66	37	56.1	97	55	56.7	137	81	59.1	380	212	55.8	0.218
NFT	197	91	46.2	180	119	66.1	135	131	97	127	110	86.6	187	140	74.9	826	591	71.5	0.871
TGN	190	176	92.6	188	178	94.7	139	134	96.4	130	123	94.6	191	188	98.4	838	799	95.3	0.759
CLN	179	166	92.7	183	178	97.3	140	133	95	129	124	96.1	177	173	97.7	808	774	95.8	0.687

*Enterobacter* and* Citrobacter* species	2015			2016			2017			2018			2019			Total			P-value*
	(*N* = 162 isolates)			(*N* = 135 isolates)			(*N* = 85 isolates)			(*N* = 124 isolates)			(*N* = 297 isolates)			(*N* = 803 isolates)			
	T	S	S%	T	S	S%	T	S	S%	T	S	S%	T	S	S%	T	S	S%	
AMP	66	32	48.5	90	40	44.4	60	54	90	90	70	77.8	184	165	89.7	490	361	73.7	0.023
AMX/CLA	39	17	43.6	36	11	30.6	60	29	48.3	66	40	60.6	83	60	72.3	284	157	55.3	0.084
CZN	41	21	51.2	58	27	46.6	51	31	60.8	118	60	50.8	159	141	88.7	427	280	65.6	0.068
CRX	58	27	46.6	78	33	42.3	78	41	52.6	120	66	55	87	71	81.6	421	238	56.5	0.003
CFX	60	21	35	70	36	51.4	77	47	61	85	46	54.1	79	53	67.1	371	203	54.7	0.801
CTM	81	33	40.7	89	44	49.4	69	53	76.8	80	59	73.8	93	67	72	412	256	62.1	0.803
CTX	41	23	56.1	89	46	51.7	80	66	82.5	100	79	79	157	129	82.2	467	343	73.4	0.548
CZM	49	24	49	102	56	54.9	83	62	74.7	81	66	81.5	107	93	86.9	422	301	71.3	0.409
CPM	88	45	51.1	128	72	56.3	80	79	98.8	122	114	93.4	151	126	83.4	569	436	76.6	0.791
IPM	132	69	52.3	130	80	61.5	85	81	95.3	120	105	87.5	180	154	85.6	647	489	75.6	0.171
MPM	141	75	53.2	131	82	62.6	84	80	95.2	119	105	88.2	221	161	72.9	696	503	72.3	0.596
PIP-TZP	120	60	50	110	67	60.9	78	61	78.2	121	116	95.9	160	135	84.4	589	439	74.5	0.180
TMP-SMZ	128	65	50.8	122	73	59.8	79	63	79.7	118	101	85.6	174	144	82.8	621	446	71.8	0.378
GMN	100	59	59	112	61	54.5	80	66	82.5	122	101	82.8	188	154	81.9	602	441	73.3	0.417
ACN	160	85	53.1	125	75	60	81	70	86.4	123	114	92.7	229	160	69.9	718	504	70.2	0.666
CIP	134	77	57.5	130	81	62.3	84	69	82.1	123	117	95.1	189	149	78.8	660	493	74.7	0.428
OXN	130	72	55.4	133	96	72.2	84	60	71.4	115	96	83.5	153	131	85.6	615	455	74	0.868
LVX	60	33	55	98	48	49	67	57	85.1	94	81	86.2	147	116	78.9	466	335	71.9	0.166
NFT	63	26	41.3	91	76	83.5	81	63	77.8	88	54	61.4	151	118	78.1	474	337	71.1	0.430
TGN	149	136	91.3	127	120	94.5	83	80	96.4	114	110	96.5	288	278	96.5	761	724	95.1	0.180
CLN	143	122	85.3	130	123	94.6	76	71	93.4	116	111	95.7	280	279	99.6	745	706	94.8	0.370

Overall	2015			2016			2017			2018			2019			Total			P-value*
	(*N* = 5,316 isolates)			(*N* = 4,705 isolates)			(*N* = 5,019 isolates)			(*N* = 5,696 isolates)			(*N* = 7,018 isolates)			(*N* = 27,754 isolates)			
	T	S	S%	T	S	S%	T	S	S%	T	S	S%	T	S	S%	T	S	S%	
AMP	1977	1170	59.2	2178	1361	62.5	2166	1370	63.3	2755	2234	81.1	3466	2835	81.8	12,542	8970	71.5	0.683
AMX/CLA	3334	2698	80.9	3244	2658	81.9	3799	3220	84.8	4246	3603	84.9	5260	4298	81.7	19,883	16,477	82.9	0.976
CZN	819	350	42.7	1999	1411	70.6	1921	1276	66.4	2271	1596	70.3	3485	2520	72.3	10,495	7153	68.2	0.375
CRX	1958	1078	55.1	2132	1256	58.9	1618	1003	62	2206	1568	71.1	3817	2568	67.3	11,731	7473	63.7	0.662
CFX	2999	1390	46.3	1679	1092	65	1952	1095	56.1	2184	1635	74.9	2304	1414	61.4	11,118	6626	59.6	0.961
CTM	928	443	47.7	960	529	55.1	1139	569	50	1345	701	52.1	1547	690	44.6	5919	2932	49.5	0.911
CTX	3829	2736	71.5	3433	2826	82.3	3185	2699	84.7	4001	3366	84.1	5724	4319	75.5	20,172	15,946	79.1	0.975
CZM	3682	2217	60.2	2565	2055	80.1	3604	2231	61.9	3178	2340	73.6	3573	2598	72.7	16,602	11,441	68.9	0.998
CPM	2696	1706	63.3	3584	3077	85.9	3988	3288	82.4	4503	3755	83.4	5310	4527	85.3	20,081	16,353	81.4	0.866
IPM	4693	3962	84.4	4347	3746	86.2	4614	4315	93.5	5357	5155	96.2	6738	6221	92.3	25,749	23,399	90.9	0.965
MPM	4511	3949	87.5	4400	3880	88.2	4538	4326	95.3	5388	5113	94.9	6814	6270	92	25,651	23,538	91.8	0.966
PIP-TZP	4347	3722	85.6	4266	3742	87.7	4561	4066	89.1	5197	4793	92.2	6255	5536	88.5	24,626	21,859	88.8	0.981
TMP-SMZ	3337	2353	70.5	3707	2838	76.6	3630	2816	77.6	4219	3516	83.3	5475	4611	84.2	20,368	16,134	79.2	0.869
GMN	4635	3808	82.2	4380	3922	89.5	4525	4079	90.1	5339	4875	91.3	6627	5929	89.5	25,506	22,613	88.7	0.967
ACN	4740	3913	82.6	4484	4235	94.4	4637	4423	95.4	5426	5156	95	6830	6513	95.4	26,117	24,240	92.8	0.960
CIP	4043	3208	79.3	3954	3374	85.3	3919	3556	90.7	5119	4211	82.3	5524	4908	88.8	22,559	19,257	85.4	0.971
OXN	1861	1186	63.7	1961	1424	72.6	1777	1274	71.7	2023	1548	76.5	2611	1703	65.2	10,233	7135	69.7	0.985
LVX	2362	1716	72.7	2560	1809	70.7	1863	1278	68.6	2679	1976	73.8	3540	2749	77.7	13,004	9528	73.3	0.847
NFT	2403	1647	68.5	3780	3039	80.4	3924	3330	84.9	4359	3698	84.8	5428	4868	89.7	19,894	16,582	83.4	0.921
TGN	4184	3782	90.4	3882	3671	94.6	3962	3767	95.1	4546	4490	98.8	6291	6022	95.7	22,865	21,732	95	0.968
CLN	3265	2859	87.6	2912	2462	84.5	2961	2688	90.8	3224	2175	67.5	6124	5887	96.1	18,486	16,071	86.9	0.608

*N* number of pathogens causing healthcare-associated infections, *T* number of tested isolates, *S* number of susceptible pathogens, *S* (%) percentage of susceptible pathogens, *AMP* ampicillin, *AMX/CLA* amoxicillin/clavulanic acid, *CZN* cefazolin, *CRX* cefuroxime, *CFX* cefixime, *CTM* cefotaxime, *CTX* ceftriaxone, *CZM* ceftazidime, *CPM* cefepime, *IPM* imipenem, *MPM* meropenem, *PIP-TZP* piperacillin/tazobactam, *TMP-SMZ* trimethoprim-sulfamethoxazole, *GMN* gentamicin, *ACN* amikacin, *CIP* ciprofloxacin, *OXN* ofloxacin, *LVX* levofloxacin, *NFT* nitrofurantoin, *TGN* tigecycline, *CLN* colistin

*Multivariate analysis of variance (MANOVA) for resistance trend

Generally, the highest susceptibilities of gram-negative pathogens to antimicrobials were seen towards: tigecycline, meropenem, imipenem and amikacin by *Escherichia coli*, 98.9%, 97.5%, 97.4% and 96.5%, respectively; amikacin, meropenem and colistin by *Klebsiella* species, 93.8%, 92.4% and 92.4%, respectively; tigecycline, colistin, amikacin and gentamicin by *Pseudomonas* species, 98.3%, 98.2%, 94.9% and 93.4%, respectively; tigecycline and colistin by *Acinetobacter* species, 95.3% and 94.6%, respectively; colistin and tigecycline by *Proteus* species, 95.8% and 95.3%, respectively; and tigecycline and colistin by *Enterobacter* and *Citrobacter* sepcies,95.1% and 94.8%, respectively.

Moreover, *Escherichia coli* was found to be highly sensitive to nitrofurantoin (94.5%), piperacillin-tazobactam (93.6%), gentamicin (92.5%) and ciprofloxacin (90.5%); against *Klebsiella* species, imipenem, amoxicillin/clavulanic acid, gentamicin and piperacillin-tazobactam retained susceptibility > 85%; *Pseudomonas* species were sensitive to ciprofloxacin (87.5%), piperacillin-tazobactam (85.2%), imipenem (83.8%), meropenem (82.9%) and ceftazidime (82.3%); *Proteus* species were sensitive to amikacin (82.5%) and meropenem (80.3%); and *Enterobacter* and *Citrobacter* species were sensitive by ≥ 70% to most of the tested antimicrobials over the 5-year period.

In contrary, lowest susceptibilities of gram-negative pathogens to antimicrobials were seen to cefotaxime and cefixime by *Acinetobacter* species, 35.2% and 37.6%, respectively. *Acinetobacter* species shown low sensitivity of ≥ 40% almost to all antimicrobials; and both *Klebsiella* and *Pseudomonas* species were slightly sensitive to cefotaxime (45% and 46%, respectively).

Over the 5 years, sensitivity of cefazolin and ofloxacin to *Pseudomonas* species increased significantly (21.5% and 31.5% increase, p-values = 0.046 and 0.030; respectively). The small sensitivity increase of *Acinetobacter* species towards tigecycline was found to be significant (0.4% increase, p-value = 0.022). In addition, large difference in susceptibility were found for both ampicillin and cefuroxime towards *Enterobacter* and *Citrobacter* species (41.2% and 35.1% increase, p-values = 0.023 and 0.003; respectively).

Prominent insignificant increase in the susceptibility of specific gram-negative bacteria to some antimicrobials occurred in 2019 compared to 2015 by: 38.4% for *Escherichia coli* to cefazolin; 30% for *Klebsiella* species to nitrofurantoin; and 65%, 52.3%, 40.6%, 33.6% and 31.5% for *Pseudomonas* species to ampicillin, cefuroxime, trimethoprim-sulfamethoxazole, cefepime and ofloxacin, respectively.

For a 5-year difference, sensitivity of *Acinetobacter* species to antimicrobials shown many insignificant increases: (rate of sensitivity increase: for cefepime, 40%; for cefotaxime, 38.1%; for levofloxacin, 36.9%; for cefixime, 36.2%; for piperacillin-tazobactam, 36.1%; for trimethoprim-sulfamethoxazole, 34.8%; for amikacin, 33.7%; and for ceftazidime, 30.2%.

*Enterobacter* and *Citrobacter* species exhibited most of the sensitivity increase changes to antimicrobials of all gram-negative isolates. In 2019 compared to 2015, *Enterobacter* and *Citrobacter* species susceptibility increased insignificantly by: 41.2% for ampicillin; 37.9% for ceftazidime; 37.5% for cefazolin; 36.9% for nitrofurantoin; 35.1% for cefuroxime; 34.4% for piperacillin-tazobactam; 33.3% for imipenem; 32.3% for cefepime; 32.1% for cefixime; 32% for trimethoprim-sulfamethoxazole; 31.3% for cefotaxime; and 30.2% for ofloxacin. However, a big insignificant decrease in susceptibility was seen in 2019 compared to 2015 by cefotaxime for *Klebsiella* species (41.4%).

Overall, among the studied antibiotics the gram-negative isolates were mostly sensitive to tigecycline (95%) whereas they were resistant to cefotaxime (49.5%) and cefixime (59.6%) (Table 2).

## Discussion

This retrospective study describes the distribution of pathogens causing HAIs and susceptibility patterns for a very high number of samples collected from both the ward and clinics in Saudi Arabia from 2015 to 2019, with an emphasis on the antibiotic classes frequently utilized to treat common infections given by a huge national surveillance program. The most commonly encountered organisms were *Escherichia coli*, *Klebsiella* species, and *Staphylococcus aureus*. Though various studies have previously described susceptibility rates in several infectious isolates, Saudi data are limited either to single-center studies [7, 12–20] or to research concentrating on the susceptibility to single or double antimicrobial classes [21–26].

One of the vital findings of the data analysis of this study was the significant increase of sensitivity for overall gram-positive bacteria to nitrofurantoin over the 5 years (30.2% increase, p-value = 0.032). Interestingly, the susceptibility of *Staphylococcus aureus* to tetracycline; MRSA to gentamicin and clindamycin; CoNS to cefoxitin, tetracycline, clindamycin, trimethoprim-sulfamethoxazole and nitrofurantoin; and *Enterococcus* species to trimethoprim-sulfamethoxazole, nitrofurantoin and vancomycin; increased insignificantly over time by ≥ 30% although this was likely due to the change of followed guidelines used for antimicrobial susceptibility testing at the Medical Group facilities, a shift from the Clinical and Laboratory Standards Institute (CLSI) to the European Committee on Antimicrobial Susceptibility Testing (EUCAST) [11, 27].

A comparison of the current results with findings from previous studies can offer some validation of the findings of this present study and identify methodological distinctions in their approaches. As expected, HAI events were more frequent in the ICUs (37.2%) compared with non-ICU locations [HAI events in wards and outpatients were 32.9% and 29.9%, respectively], a finding which was previously described in local studies [10, 28] and may reflect the epicenter role of ICU in both infections and antimicrobial resistance. The predominant isolates to cause HAIs were gram-negative organisms (71.9% vs. 28.1%); this finding was similar with many Saudi studies made in different cities in Saudi Arabia including Riyadh [6, 28, 29], Makkah [30, 31], Dhahran [32], Bisha [33], and Aljouf [10]; with the majority being *Escherichia coli* (38%) accounting approximately for 52.9% of the gram-negative bacterial growth in line with previous national studies [7, 29, 30, 32, 34]. The second predominant isolates of the gram-negative organisms were the *Klebsiella* species (15.1%), this finding was similar to the bacterial isolates prevalence studies from Dhahran [7], and Riyadh [6]. The proportions of *Klebsiella* were 17.2% in Dhahran [7], and 14.7% in Riyadh [6]. The culture rate in our study for *Proteus* species (2.3%) was comparable to previously reported rates in two different studies in Riyadh (1.2% and 1.8%) [6, 35]. Also, the incidence of *Acinetobacter* species in our study was very close to the rate reported before (4.2% vs 5.5%) [6]; in contrast to the much higher rates found in two separate studies in Riyadh (31.7% and 25.3%) [35, 36]. Our prevalence of *Staphylococcus aureus* was similar to the rate described in a previous report done in Riyadh (12.6% vs 13.9%) [6]. We report a lower rate of MRSA (15.9%) compared to two previous studies made in Riyadh (24.4% and 30.3%, respectively) [28] but similar to the rate reported before in another study in Riyadh (17.5%) [29]. We report a higher susceptibility of *Enterococcus* species to vancomycin (85.9% vs 79.7%) compared to one study in Riyadh [6]. In our study, proportion of *Pseudomonas* species that caused HAIs is less than what was reported in Riyadh (10.1% vs 15.4%) [6]; however, our prevalence was in agreement to the bacterial frequency in a study from Dhahran (12.8%) [7]. Frequency of CoNS in causing HAIs in this study is in line with a study from Riyadh (5.2% vs 6.5%) [6] but much lower than the rate reported previously in a study in Riyadh (28.4%) [29]. In our study, incidence of *Enterococcus* species as causative pathogens for HAIs is almost half of the reported rate by a study in Riyadh (4.5% vs 8.6%) [6]; however, rate was in parallel to the prevalence reported in other study in Riyadh (5.9%) [28] but contradicts with the rate reported in another study in Riyadh (15.8%) [29].

Our data analysis regarding the susceptibility patterns of antimicrobials confirm or contradict the findings of previous local studies. For example, *Pseudomonas* and *Acinetobacter* were most susceptible to colistin and amikacin in a study in Riyadh [29], whereas in our study, tigecycline and colistin had higher susceptibility rates. On the other hand, *Escherichia coli*, *Klebsiella pneumonia*, *Enterobacter* and *Citrobacter* species were most sensitive to amikacin, imipenem and meropenem [29], whereas in our study, *Escherichia coli* and *Enterobacter* and *Citrobacter* species were most susceptible to tigecycline, and *Klebsiella* species was most susceptible to amikacin. However, our study support the finding that CoNS were most susceptible to vancomycin and linezolid [29] and we found the susceptibility of *Staphylococcus aureus* to clindamycin and trimethoprim-sulfamethoxazole were almost identical to the results of the aforementioned study (92% vs 94% and 87.7 vs 87%, respectively). This is might be due the fact that the sample was drawn from tertiary private hospitals in Saudi Arabia where the level of environmental hygiene is higher and staff are highly restricted to infection control practices.

Linezolid and vancomycin had the best susceptibility profile to *Staphylococcus aureus*, CoNS, and *Enterococcus* species while gentamicin shown low sensitivity towards MRSA, CoNS and *enterococcus* species. In the context of emergence of resistance of malicious gram-positive bacteria to gentamicin, linezolid and vancomycin have become effective alternatives to gentamicin treatment frequently associated with nephrotoxicity [37]. Linezolid and vancomycin are active against the most serious gram-positive bacteria, including *streptococci*, vancomycin-resistant *enterococci* (VRE) and MRSA [38]. Nevertheless, we noted a low rate of susceptibility of linezolid and vancomycin against MRSA (54.9% and 50.9%, respectively) likely because of antibiotic selection pressure and possibly a reflection of selective reporting of susceptibility testing; this finding contradicts those of a recent study in Riyadh, which identified a 100%-sensitivity of both antimicrobial agents for MRSA [29]. The relatively lower susceptibility in gram-positive bacteria in the current study may be reflecting Saudi prescription trends in recent years that overuse fluoroquinolones [1, 39] and carbapenems [40, 41] at the expenses of other broad-spectrum such as linezolid and vancomycin due to increased availability and reduced cost of these drugs. However, nitrofurantoin maintained the greatest efficacy against MRSA in our study (82.6%); supporting the finding of a recent study in Aseer that shown 100% susceptibility of MRSA to nitrofurantoin [42].

Over the 5-year period, it is interesting to note imipenem and meropenem either retained its activity or shown susceptibility increase patterns towards all the studied gram-negative pathogens except for imipenem which was less sensitive in 2019 by 1.6% against *Escherichia coli* and for meropenem that shown a minor sensitivity reduction by 8.6% to *Klebsiella* species. Previous studies from Saudi found high susceptibility of *Pseudomonas* to carbapenems [7, 41]; however, in other local studies, the susceptibility of *Pseudomonas* to meropenem declined over a five-year period [29] and nonsusceptibility of *Acinetobacter* and *Pseudomonas aeruginosa* to carbapenems was very high (68.3% and 76%) [6, 41]. Furthermore, there were relatively stable susceptibility patterns to all tested antimicrobials, except for cefotaxime which shown a susceptibility reduction by 41.4%, among *Escherichia coli* and *Klebsiella* species; in opposite to the finding of a local study in Dhahran that shown a reduction trend in the susceptibility of antibiotics to *Escherichia coli* and *Klebsiella* species [7]. Moreover, we observed an increase in the susceptibility of *Acinetobacter* and *Enterobacter* and *Citrobacter* species to all studied antimicrobials except for colistin that had a slight sensitivity reduction by 4.3% against *Acinetobacter* species. This can be considered as a part of the success of the combating strategies implemented since January 2014 at the medical settings to reduce further emergence and spread of AMR, lower the percentage of HAIs and MDR organisms, and save on needless healthcare expenses [1].

Significant differences in antibiogram findings between different healthcare facilities and regions may suggest differences in populations of the served patients, patterns of antimicrobial use, or deficiencies in hospital infection control and hygiene practices that could be further explored.

## Limitations

This study had a few limitations. Firstly, the retrospective design and the risk of misclassification and selection bias. For instance, even though the laboratories follow the highest standards, there may be a possibility that some isolates had some contaminants. Furthermore, since all three hospitals in this study are tertiary care hospitals, they receive more complicated cases that may be caused by resistant pathogens which may not indicate the antibiotic susceptibility trend and microbiology of the general population. Nevertheless, our study’s findings will add to local and global data on antimicrobial susceptibility, especially with highly threatening infections.

## Conclusion

Systematic collection and analysis of routine clinical laboratory data is important in assessing the antimicrobial resistance burden. Nationwide surveillance is urgently needed to provide policy makers, antimicrobial stewardship committees, infection preventionists, microbiologists, and epidemiologists with essential information to guide proper action plans. The observed increase in susceptibility of gram-positive and gram-negative bacteria to studied antimicrobials is important; however, reduced sensitivity of MRSA, CoNS and *Enterococcus* species to gentamicin; and increased resistance of MRSA to linezolid and vancomycin is a serious threat and calls for effective antimicrobial stewardship programs.

## Data Availability

Data are available upon request, please contact author for data requests.

## Abbreviations

AMS: Antimicrobial Stewardship
HAIs: Healthcare Associated Infections
MRSA: Methicillin-resistant *Staphylococcus aureus*
VRE: Vancomycin-resistant *Enterococci*
CoNS: Coagulase-negative *Staphylococci*
CLABSI: Central line associated bloodstream infection
CAUTI: Catheter-associated urinary tract infection
SSI: Surgical site infection
VAE: Ventilator-associated events
HMG: Habib Medical Group
AMR: Antimicrobial resistance
MANOVA: Multivariate analysis of variance
ICUs: Intensive care units
CLSI: Clinical and Laboratory Standards Institute
EUCAST: European Committee on Antimicrobial Susceptibility Testing
ATCC: American Type Culture Collection
